# Nondestructive Detection of the Gel State of Preserved Eggs Based on Dielectric Impedance

**DOI:** 10.3390/foods10020394

**Published:** 2021-02-11

**Authors:** Cheng-Han Li, Chun-Hung Hsieh, Cheng-Chu Hung, Ching-Wei Cheng

**Affiliations:** 1 Department of Bio-Industrial Mechatronics Engineering, National Chung Hsing University, Taichung 402, Taiwan; cghan@smail.nchu.edu.com.tw (C.-H.L.); ackt223@gmail.com (C.-C.H.); 2 Department of Information Management, National Taichung University of Science and Technology, Taichung 404, Taiwan; guu@nutc.edu.tw; 3 College of Intelligence, National Taichung University of Science and Technology, Taichung 404, Taiwan

**Keywords:** preserved egg, nondestructive, dielectric method, impedance

## Abstract

After completing the production of preserved eggs, traditionally, the degree of gelling is judged by allowing workers to tap the preserved eggs with their fingers and sense the resulting oscillations. The amount of oscillation is used for the quality classification. This traditional method produces varying results owing to the differences in the sensitivity of the individual workers, who are not objective. In this study, dielectric detection technology was used to classify the preserved eggs nondestructively. The impedance in the frequency range of 2–300 kHz was resolved into resistance and reactance, and was plotted on a Nyquist diagram. Next, the diagram curve was fitted in order to obtain the equivalent circuit, and the difference in the compositions of the equivalent circuits corresponding to gelled and non-gelled preserved eggs was analyzed. A preserved egg can be considered an RLC series circuit, and its decay rate is consistent with the decay rate given by mechanical vibration theory. The Nyquist diagrams for the resistance and reactance of preserved eggs clearly showed that the resistance and reactance of gelled and non-gelled eggs were quite different, and the classification of the eggs was performed using Bayesian network (BN). The results showed that a BN classifier with two variables, i.e., resistance and reactance, can be used to classify preserved eggs as gelled or non-gelled, with an accuracy of 81.0% and a kappa value of 0.62. Thus, a BN classifier based on resistance and reactance demonstrates the ability to classify the quality of preserved egg gel. This research provides a nondestructive method for the inspection of the quality of preserved egg gel, and provides a theoretical basis for the development of an automated preserved egg inspection system that can be used as the scientific basis for the determination of the quality of preserved eggs.

## 1. Introduction

Preserved eggs are mainly produced by marinating fresh eggs. Marinated duck eggs are much easier to store than fresh duck eggs, and they contain large amounts of protein and amino acids [1]. Preserved eggs exhibit a unique smell and taste that can enhance the appetite, and they are well known in Asia as a traditional Chinese food. Famous preserved egg dishes—such as tofu with preserved egg, three cups of preserved egg, and lean porridge with preserved egg—are very popular. Preserved eggs are also exported to other countries, such as the United States, Canada, and Japan, where their unique aroma is appreciated. According to statistics from the Taiwan government and the Duck Association of the Republic of China, Taiwan’s annual output of duck eggs is approximately 460 million, and the yearly total value is approximately US $56 million. The majority of the duck eggs are converted into processed duck eggs. The preservation of the eggs depends on pickling using alkaline substances. This causes chemical reactions involving the proteins inside the eggs, and results in their solidification. In general, the methods of producing preserved eggs can be divided into three categories:

- Coating method: The fresh eggs are wrapped in a strong alkaline mud consisting mainly of a mixture of plant ash, white lime, and rice husks, and then they are kept at room temperature for 30–40 days [2].
- Immersion method: This is currently the primary method of making preserved eggs. Salt, tea, and other substances are mixed with sodium hydroxide or potassium hydroxide in particular proportions. In order to adjust the osmotic pressure of the alkaline solution in the egg, and to improve the success rate of the process, minute amounts of copper, zinc, iron, and metal salt compounds are added to the pickling liquid. Using this method, the time taken to produce the preserved eggs depends on the concentration of the alkaline solution, the type and concentration of metal compounds used, and the immersion temperature. Generally, in this way, preserved eggs can be produced in a shorter time than using traditional methods that work at room temperature [3,4].
- Combined method: This method involves a combination of the coating and immersion methods. The fresh eggs are wrapped in paper and put into a plastic bag, and a small amount of alkaline solution is added. The paper with the alkaline solution indirectly immerses the egg in the alkaline liquid, and then the egg is left at room temperature for 30–40 days to mature [2].

Because of the interaction between the duck egg and these alkaline substances, the duck egg becomes a preserved egg. The shell of a standard preserved egg is easy to remove, and the appearance of the preserved egg’s albumen is smooth, and similar to that of tan jelly. Patterns shaped like pine needles or needle-like crystals are formed as the egg yolk moves to the egg albumen. The egg yolk becomes solid, and typically appears dark green or grass green. If the gel of the preserved egg is incomplete, a thick, brown liquid may flow out when the eggshell is removed; the eggshell may also stick to the surface of the albumen and be difficult to remove. Such eggs also emit a pungent smell, which may cause nausea. The mature preserved eggs are removed from the soaking bucket and dried. After drying, the quality of the preserved eggs is manually determined by tapping the eggs and sensing the amount of oscillation. Preserved eggs that are gelled to the normal standard oscillate strongly when they are tapped with a finger; this is the primary detection method that is currently used in preserved egg factories. The use of this method may cause injury to factory workers. Eventually, the workers can lose sensitivity in their fingers, which also leads to misclassification. No standard way of quantifying the degree of gelling of a preserved egg exists; therefore, long-term experience is needed to identify the degree of gelling using the tapping method.

The quality of many agricultural products is estimated by measuring their electrical characteristics. For example, one method involves inserting steel needles into fruit tissue that becomes an electrode; the voltage and frequency are then varied, and the change in the electrical impedance is measured. Thus, changes in the internal structure of different varieties of apples during storage can be estimated from the changes in the impedance at different frequencies [5]. The internal moisture content of the yolk and protein in poultry eggs changes with time. In order to determine their storage time [6], the moisture content can be determined by measuring the electrical conductivity using an alternating-current voltage, which is a destructive test. In the case of nondestructive methods, most of the dielectric parameters are measured using capacitive methods or open-circuit terminal probes. Ghaderi et al. (2018) used a nondestructive method employing parallel plate capacitors to measure the dielectric constant and loss factor by changing the voltage frequency to classify poultry eggs as fertilized, nonfertilized, or dead [7]. The nondestructive testing of eggs using parallel plate capacitors, changing the frequency range of the sine wave voltage within the range of 40 kHz–20 MHz, and measuring the eggs’ dielectric parameters can be used to predict the freshness of poultry eggs [8]. Other studies, in which dielectric properties were used to determine the quality of different products, involved fruits [9], agricultural products [10], and foods [11]. However, research on the detection of preserved egg gel is scarce. In one study, Chen et al. (2011) proposed the use of the plastic stick-tapping method to determine the quality of gelling in preserved eggs from which the shell was not removed [12]. The preserved eggs were tapped using a constant force, and an accelerometer was used to determine the amount by which the eggs oscillated. The rate at which the oscillations decayed was low in the case of gelled eggs, and high if the eggs had not been gelled. The corresponding changes in the electrical impedance of the biological samples may vary according to the frequency of the external electrical field, with tissue structure and composition differences [13,14]. Therefore, the electrical parameters from fitting circuits can be conveniently explored by impedance, resistance, and reactance in order to describe the samples’ response and determine the preserved eggs’ state.

The purpose of this research was to investigate a nondestructive dielectric method to determine whether the gelling of preserved eggs is complete. The classification of eggs as either gelled or non-gelled preserved eggs was based on their dielectric impedance. This method could possibly replace the traditional method of tapping the preserved eggs with fingers to classify the gel quality. The difference between the results that were obtained based on the detection of the dielectric properties and those obtained using the tapping method [12] was also investigated. Equivalent circuits for completely and incompletely-gelled preserved eggs were established, and dielectric impedance was used to classify the gelling quality of the preserved eggs.

## 2. Materials and Methods

### 2.1. Samples

**Figure 1:**
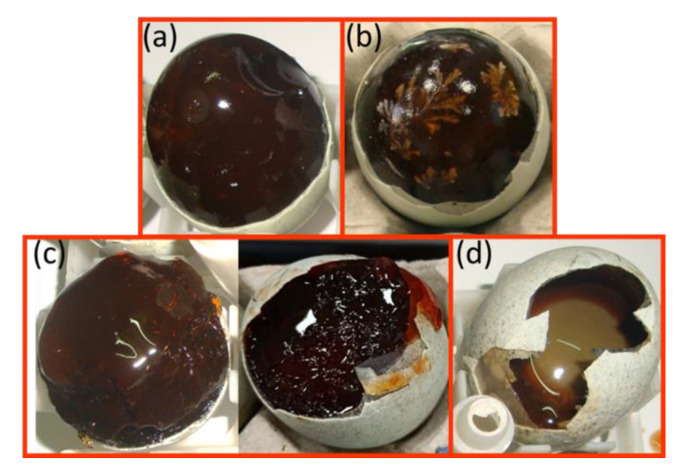
Preserved eggs after the removal of the shell: (**a**) a gelled egg and (**b**) the small end of a gelled egg showing the ‘pine flower’ pattern in the albumen; (**c**,**d**) viscid and liquid non-gelled eggs, respectively.

The samples of gelled and non-gelled preserved eggs used in this study were produced by the Chuan-ming egg company based in Chiayi County, Taiwan. The preserved eggs were all produced from fresh duck eggs by immersing them in an alkaline solution. All of the preserved eggs were produced in the same production conditions following the same procedures. The gelled and non-gelled preserved eggs were produced by marinating a large number of preserved eggs. After the production of the preserved eggs was complete, the factory workers tapped them to determine their gel status. In this study, the eggs shown in Figure 1a,b were defined as completely-gelled preserved eggs. When these shells were removed, the albumen at the small end of the preserved eggs was elastic and smooth, and no thick liquid was present; the albumen had also completely solidified. Figure 1b shows high-quality preserved eggs that exhibit ‘pine flowers’ in the egg whites. This type of preserved egg is called a ‘pine flower preserved egg.’ Figure 1c,d shows non-gelled preserved eggs that contained a thick liquid or liquid albumen at the small end after the shell was removed. In this study, the eggs shown in Figure 1c,d were defined as non-gelled preserved eggs, or incompletely-gelled preserved eggs.

In this study, we considered 176 samples of gelled and non-gelled preserved eggs. The impedance and loss factors of the preserved eggs were measured using an LCR-6300. After all of the samples were investigated, the eggs were first classified as gelled or non-gelled by Bayesian network (BN) classification in machine learning, and 30 gelled preserved eggs and 30 non-gelled preserved eggs were used for the training of the machine-learning model. In addition, 116 preserved eggs were used for the classification accuracy verification.

### 2.2. Impedance Spectrum Measurements

**Figure 2:**
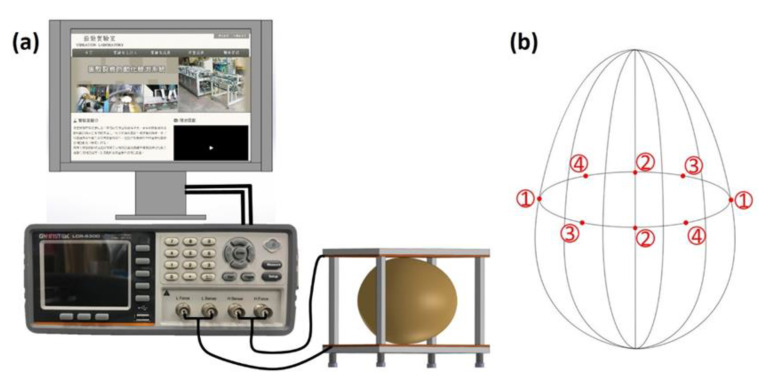
(**a**) Schematic of the measurement setup; (**b**) positions where the parallel plate capacitor was placed during the measurements.

The measurements of the preserved eggs were made indoors at an average temperature of 25 °C and a relative humidity of 43%. The impedance spectrum measurements were taken using an LCR-6300 (Good Will Instrument Co., Ltd., New Taipei City, Taiwan) with homemade parallel plate capacitors. The LCR-6300 is generally used to measure the resistance, inductance, and capacitance of electronic components, and its output and measurement frequency range are 10–300 kHz. The LCR-6300 was used to measure the impedance of the preserved eggs using parallel plate capacitors. Figure 2a shows the measurement setup. The parallel plate capacitor was comprised of two pieces of acrylic (75 × 55 mm), two pieces of conducting-cloth–covered foam (60 × 55 mm) (U-TEK EMI Corp., New Taipei City, Taiwan), and four springs. The conducting foam was compressible, which meant that the contact area and the tightness of the contact between the conducting plane and the preserved egg could be increased. The four springs in the parallel plate capacitor allowed the distance between the parallel plates to be automatically adjusted according to the size of the egg. The parallel plate capacitor is shown in Figure 2a. The measurements were made on individual eggs without removing the shell. Each pair of numbers (1–4) in Figure 2b represents one pair of contact positions of the parallel plates; the data from the four different pairs of positions were averaged after the measurements were taken.

### 2.3. Dielectric Analysis

The dielectric substance generates an external electric field, the internal charges in the material move in a given direction under the action of the electric field, and the charge is redistributed over the surface of the dielectric material. Then, electrons inside the dielectric cannot move freely, which means that the dielectric substance does not conduct electricity under normal circumstances; however, a dielectric material is not necessarily an insulator. When the gap between two conducting electrodes is filled by a dielectric, more energy is stored than would be stored in the absence of the dielectric. The dielectric constant can be used to express the ability of a material to store energy. The dielectric constant is not a fixed value; it depends on factors such as the frequency, temperature, and molecular structure of the material.

The change in the capacitance of a capacitor mainly depends on the dielectric constant, $\varepsilon^{\prime }$, of the material between the two electrode plates, as well as the geometry, size, and relative positions of the two electrodes. Equation (1) is the basic capacitor equation:

(1) $$C=\frac{\varepsilon^{\prime }\varepsilon_{0}S}{d}$$

where C is the capacitance (units: Farad (F)), $\varepsilon^{\prime }$ is the relative permittivity, $\varepsilon_{0}$ is the vacuum permittivity (8.85 ×10−12 Fm), *S* is the plate area $\left(m^{2}\right)$, and d is the distance between the two plates (m).

The dielectric properties will change as the frequency of the applied alternating voltage changes. Because of the interaction between the external electric field and the sample, the dielectric constant will also change. The electrical impedance (*Z*) under the alternating voltage is composed of a real part—the resistance, *R*—and an imaginary part—the reactance, *X*—and can be expressed by Equation (2). The relationship between the alternating voltage and the current depends on the impedance and phase angle of the impedance, θ; the resistance and reactance are given by Zcosθ and Zsinθ, respectively [15]:

(2) Z=R+jX

and

(3) $$Z=Ze^{j\theta }=Z\;(\cos\theta +j\sin\theta )$$

The loss angle, *δ*, is the same as the angle (*θ*) between the voltage and the current, but with a phase difference of 90°. The loss factor can also be expressed in terms of tanδ (Equation (5)):

(4) $$\delta =\frac{\pi }{2}-\theta$$

and

(5) $$Loss\;factor\left(D\right)=\frac{R}{X}=\tan\delta$$

### 2.4. RLC Resonance Circuit Analysis

Resistance, inductance, and capacitance can be connected in series to form a circuit. An RLC circuit such as this produces damped oscillations, and it can be represented by the RLC resonance circuit equation:

(6) $$L\frac{d^{2}i}{dt^{2}}+R\frac{di}{dt}+\frac{1}{C}i\;=\;0$$

the solution to which can be written as

(7) $$i\left(t\right)=A_{1}e^{-\omega_{0}\left(\zeta +\sqrt{\zeta^{2}-1}\right)t}+A_{2}e^{-\omega_{0}\left(\zeta -\sqrt{\zeta^{2}-1}\right)t}$$

When the damping factor *ζ* < 1, the system is termed an under-damped system, which can also be represented by

(8) $$i\left(t\right)=Ce^{-\alpha t}\cos\left(\omega_{d}t+\theta \right)$$

where *α* is the RLC series circuit. The rate of attenuation of the oscillations in the current during the transient response can be written as

(9) $$\alpha =\frac{R}{2L}$$

### 2.5. Statistical Analysis

A BN classifier is a probabilistic graphical model. A directed acyclic graph is composed of a directed arc that represents the probability condition relationship between the node representing the variable and the target node. The nodes in the structure represent a set of random variables, *X* = *X*1, …, *Xn*, and the directed edges connecting the two nodes represent the dependencies between the variables [16]. Given the graph structure and variable data set, a conditional probability table can be established, which shows the degree of dependence between the variables. In this study, the BN classifier in the WEKA 3.8 software was used to classify the gelling quality of the preserved eggs based on the impedance, resistance, reactance, and decay rate of an RLC circuit. The BN search employed the k2 algorithm.

The confusion matrix and receiver operating characteristic (ROC) curve are indicators of the effectiveness of classification results. These methods are often used in clinical medical diagnosis and binary data judgment related to the diagnosis of diseases [17,18]. In this study, the preserved eggs were divided into two categories: completely gelled and incompletely gelled. The shells of the preserved eggs were removed after the measurements were completed. Then, the results were compared with the real gelling state for the classification. In the case of completely gelled eggs, if the results of the measurements agreed with the real status of an egg, then the result was classified as TP (true positive). For incompletely-gelled eggs, if the measurements also determined that an egg was incompletely gelled, the result was classified as TN (true negative). If the results of the measurements showed that an egg was completely gelled, but in reality, the egg was not completely gelled, the result was classified as FP (false positive). Finally, if an egg was completely gelled, but the results of the measurements showed that it was incompletely gelled, the result was classified as FN (false negative). These four possibilities can be expressed as a 4 × 4 confusion matrix [19] (Metz, 1978), as given in Table 1. Using this table, the accuracy (10) and precision (11) of the dielectric measurement technique could be determined.

(10) $$\mathrm{Accuracy}=\frac{\mathrm{TP}+\mathrm{TN}}{\mathrm{TP}+\mathrm{TN}+\mathrm{FP}+\mathrm{FN}}\;\;$$

(11) $$\mathrm{Precision}=\frac{\mathrm{TP}}{\mathrm{TP}+\mathrm{FP}}\;$$

(12) $$\mathrm{Sensitivity}=\frac{\mathrm{TP}}{\mathrm{TP}+\mathrm{FN}}$$

In graphs showing the ROC, the sensitivity is plotted on the y-axis and the specificity is plotted on the x-axis. The area under the curve in such a graph is called the area under the curve of the ROC (AUC ROC). The AUC index is used to evaluate the accuracy of the classification, and it exhibits a value between 0 and 1. The larger the value of this index, the more accurate the classification. In the case of random guessing with two choices, the value will not be lower than 0.5; as such, in the case of a real classifier, the AUC will also not be less than 0.5 [20]. Cohen’s kappa [21], which is another indicator used for the evaluation of the quality of classifications, is defined according to Equation (13). If the value of this indicator is less than 0.40, the classification lacks reliability and consistency. Values of 0.41–0.60 indicate that the classification is moderately reliable and consistent, and values of 0.61–0.80 indicate reliable classification with excellent consistency. A value greater than 0.80 means that the classification is almost perfect [22]. The definition of Cohen’s kappa is

(13) $$\kappa =\frac{p_{0-}p_{c}}{1-p_{c}}$$

where $p_{0}=\frac{\mathrm{TP}+\mathrm{TN}}{\mathrm{TP}+\mathrm{FP}+\mathrm{FN}+\mathrm{TN}}\;\mathrm{and}\;p_{c}=\frac{\left(\mathrm{TP}+\mathrm{FP}\right)\left(\mathrm{TP}+\mathrm{FN}\right)+\left(\mathrm{FN}+\mathrm{TN}\right)\left(\mathrm{FP}+\mathrm{TN}\right)}{{\left(\mathrm{TP}+\mathrm{FP}+\mathrm{FN}+\mathrm{TN}\right)}^{2}}$.

## 3. Results and Discussion

### 3.1. Impedance Spectroscopy Analysis

**Figure 3:**
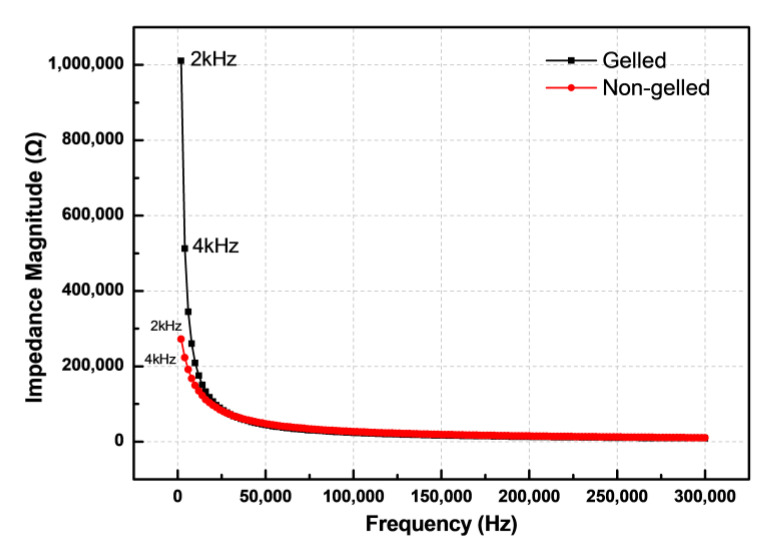
Plots of impedance against frequency for completely-gelled and incompletely-gelled preserved eggs.

In this study, an LCR meter and parallel plate capacitors were used to measure the impedance spectra of completely-gelled and incompletely-gelled preserved eggs between 2 and 300 kHz. Figure 3 shows that the impedance of gelled and non-gelled preserved eggs decreases with an increase in the frequency. Moreover, at the same frequency, eggs that are not completely gelled exhibit lower impedance than completely-gelled eggs. The impedance is composed of real resistance and imaginary reactance. In this section, the resistance and reactance of completely- and incompletely-gelled preserved eggs will be analyzed.

**Figure 4:**
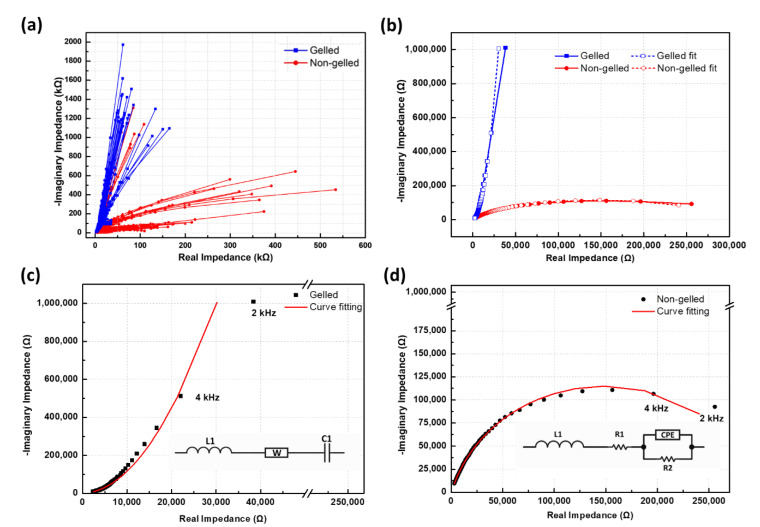
(**a**) Nyquist plots (impedance curves) for 30 completely- and 30 incompletely-gelled preserved eggs. (**b**) Nyquist plots from Figure 4. (**a**) Two fitted impedance curves for completely- and incompletely-gelled preserved eggs. Also shown are the fitted impedance curves (range: 2–300 kHz) obtained by simulating the equivalent circuit of (**c**) completely-gelled and (**d**) incompletely-gelled preserved eggs.

Figure 4b reveals significant differences between the Nyquist plots of completely- and incompletely-gelled preserved eggs. Figure 4a shows that the reactance of gelled-preserved eggs and incompletely-gelled preserved eggs are both negative in the range of 2–300 kHz, which indicates that both gelled- and incompletely-gelled preserved eggs exhibit capacitive characteristics. In Figure 4, the Nyquist plot of the reactance versus the resistance of completely-gelled preserved eggs in the range of 2–300 kHz gives an oblique line. The inside of a preserved egg that is completely gelled is solid, and the charged ions inside the preserved egg will separate under the action of an electric field. However, because the inside of the egg is solid, the charged ions cannot move to the positive or negative pole, so the Nyquist plot of the reactance and resistance produces an oblique straight line. This process represents diffusion effects. The Nyquist plot of incompletely-gelled preserved eggs is a semicircle, as shown in Figure 4. Under the action of an electric field with a frequency between 2 and 300 kHz, the internal ions of incompletely-gelled preserved eggs will quickly separate. Because the ions inside the liquid interior of incompletely-gelled preserved eggs can easily move to the electrodes at both ends, the charge transfer process will appear as a semicircular arc in the Nyquist plot. The preserved eggs’ measurement frequency range of 2–300 kHz corresponds to beta dispersion. The beta dispersion frequency associated with the polarization of cellular membranes, protein, and other organic macromolecules ranges from 1 kHz to several MHz [23]. The impedance of gelled and non-gelled preserved eggs will change, as shown in Figure 4. The inference is that the degree of albumen gelation in preserved eggs is different.

The capacitive characteristics of real electronic components can be used to analyze completely- and incompletely-gelled preserved eggs. At low frequencies, the impedance and reactance of incompletely-gelled preserved eggs will be lower than those of completely-gelled preserved eggs. By contrast, the resistance of incompletely-gelled preserved eggs is greater than that of completely-gelled preserved eggs. Normal capacitors tend to block direct current while allowing alternating current to pass. In the case of a low-frequency alternating current, the resistance of a capacitor will be relatively large, thus preventing direct current from passing, whereas the reactance will be small, allowing alternating current to pass.

In terms of electronic characteristics, an incompletely-gelled preserved egg is similar to a capacitor. Compared with a real capacitor, the resistance of resistor R2, which is shown connected in parallel to the constant phase element (CPE) in Figure 4d, will be very large. R2 can be regarded as a bleeder resistor, and it quickly consumes the electrical energy in the preserved egg, which means that it does not exhibit the ability to store energy in the same way as an electronic capacitor. Compared with incompletely-gelled preserved eggs, the electrical resistance of completely-gelled preserved eggs is minimal, and the reactance is large. In the case of real electronic capacitors, when the electrolyte inside the capacitor is lost or has dried up, the capacitor will form a short circuit. Then, the internal resistance will be very small, and the reactance will be large; this will cause the capacitor to heat up and suffer damage when an alternating-current voltage passes through it. In a completely-gelled preserved egg, no watery albumen is present, which means that the resistance is minimal under a low-frequency alternating-current voltage; therefore, the reactance is very large, which is similar to the case of a capacitor with no electrolyte inside.

The gelling of preserved eggs is a process of continuous and gradual change, and the reactance and resistance of preserved eggs changes during this process. In the present study, the researchers speculated that the reactance of incompletely-gelled preserved eggs would be lower than that of completely-gelled preserved eggs. The reason for this is that the inside of incompletely-gelled preserved eggs consists of liquid albumen; therefore, the ions inside the egg can move easily under the influence of alternating current, giving a small reactance value. After the same marinating time, both completely-gelled and incompletely-gelled preserved eggs can be produced. This is because the gelling may fail, owing to the excessive alkalinity of the pickling liquid or the excessive absorption of the liquid by the egg during the pickling process. The albumen inside preserved eggs that are excessively alkalized will become liquid, and the reactance will be low.

According to the National Standard of the Republic of China, CNS 2174 (Taiwan, 1986), regarding testing methods for alkalized eggs, the albumen at the small end of preserved eggs should have liquefied and be sticky. The yolk should solidify, and if the conditions during the pickling process are excessively alkaline, the gelling process can fail or be incomplete. The characteristics of an incompletely-gelled preserved egg include the albumen failing to gel, and a solidified egg yolk. According to the CNS 2174 guidelines, such an egg will be considered over-alkalized, or the consideration will be that the pickling liquid was too alkaline. The Nyquist plots for completely- and incompletely-gelled preserved eggs shown in Figure 4c,d, respectively, were analyzed and simulated using noncommercial impedance spectroscopy software in order to establish the equivalent circuits for the two different states of preserved eggs. These equivalent circuits were series circuits comprising resistance, inductance, and capacitance. The circuits were also similar to the equivalent circuits for electronic capacitors.

Element *W* in Figure 4c is known as the Warburg element, which is composed of the diffusion admittance (*Y*_0_) and the diffusion time constant (*B*). These components are related to changes in the Warburg impedance, as given by Equation (14):

(14) $$Z_{w}=\frac{1}{Y_{0}}tanh\left(Bj\omega \right)$$

where *ω* is the angular frequency. When the admittance value, *Y*_0_, increases, the total impedance decreases and the diffusion time constant, *B*, decreases. This may be due to the higher concentration of charged ions in the medium, which increases the total conductivity, affects $Y_{0}$, and reduces the value of *B*.

The admittance of the CPE shown in Figure 4d can be calculated using Equation (15):

(15) $$Y_{CPE}=\frac{1}{Z_{CPE}}=Q_{0}{\left(j\omega \right)}^{n}$$

Figure 4c shows the equivalent circuit element after the curve fitting, in which L1 = 218 pH. The Warburg impedance elements used were 4.786 $\frac{M\Omega }{\sqrt{s}}$; C1 is a capacitor with a capacitance of 81.67 pF. Figure 4d shows the values of the components of the equivalent circuit after the curve fitting. L1 is an inductor with an inductance of 583.7 µH, resistor R1 exhibits a resistance of 46.25 Ω, resistor R2 exhibits a resistance of 298.3 kΩ, and the value of $Q_{0}$ for the CPE is 503.5 ${\mathrm{ps}}^{n}\cdot \Omega^{-1}$, where *n* is 0.8361. When *n* = 1, the CPE is an ideal capacitor; when *n* = −1, it is an inductor; and when *n* = 0, it is a pure resistor. When *n* > 0.8, the CPE demonstrates the characteristics of a capacitor.

To summarise, we can regard a preserved egg as a capacitor. A fully-gelled preserved egg has small resistance and large reactance, similar to a bad capacitor in which the electrolyte is dried up, as shown in Figure 4c. An incompletely-gelled preserved egg has a large resistance and small reactance, similar to a normal capacitor with the presence of an electrolyte inside, as shown in Figure 4d. Therefore, the impedance measurement was conducted under the AC signal at the same frequency, and the impedance of a fully-gelled preserved egg was greater than an incompletely-gelled preserved egg, as shown in Figure 3.

### 3.2. Analysis of the Decay of the Circuit Transient Response

Chen et al. (2011) measured the rate of decay of the oscillations of a preserved egg by tapping a preserved egg and judging the quality of the preserved egg’s gel from the decay rate [12]. The rate of decay of the oscillations for a completely-gelled egg was smaller than that for an incompletely-gelled preserved egg.

In Section 3.1 of this paper, the researchers described how the impedance of preserved eggs was used to simulate the equivalent circuits for completely- and incompletely-gelled preserved eggs, which are shown in Figure 4c,d, respectively. The eggs here are equivalent to a series combination of a resistor, capacitor, and inductor, which can be expressed as Equation (6). The equation for an RLC resonance circuit (Equation (6)) is similar to the equation for a damped mechanical oscillation. The transient response exhibits an oscillation-signal decay rate of α, which can be calculated using Equation (9).

In order to obtain the resistance, capacitance, and inductance values of the preserved egg, the resistance, inductance, and capacitance of the RLC resonance circuit were measured using an LCR meter. The measured inductance value of the LCR meter was negative. The main reason for this was that the reactance was negative. This can be deduced from Equation (16) which gives the inductance:

(16) L=X/2πf

where $X=X_{L}-X_{c}$, L is the inductance, X is the total reactance, f is the frequency, $X_{L}$ is the inductive reactance, and $X_{c}$ is the capacitive reactance.

**Figure 5:**
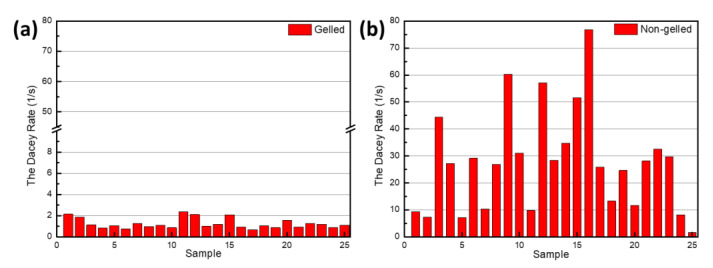
Rates of current decay at 6 kHz for (**a**) completely-gelled and (**b**) incompletely-gelled preserved eggs.

The reactance measured using the LCR meter was the total reactance. The capacitive reactance of a preserved egg is greater than its inductive reactance, which means that the total reactance is negative. The inductance is also negative, and a preserved egg exhibits relatively few inductive components. As the inductance value becomes very small at a frequency of 6 kHz, the inductance, capacitance, and resistance measured at a frequency of 6 kHz were used in the equation for the RLC resonance circuit. Figure 5 shows the rate of energy attenuation for the transient response of the RLC circuit at a frequency of 6 kHz; Figure 5a,b correspond to completely- and incompletely-gelled eggs, respectively. It can be seen that the decay rate for completely-gelled eggs is less than ~2.3 $s^{-1}$, whereas the average decay rate of incompletely-gelled preserved eggs is more than ~8 $s^{-1}$. Thus, a significant difference exists between these two rates.

The amount of energy stored in completely-gelled preserved eggs decays slowly, whereas the energy stored in incompletely gelled eggs decays quickly. It can be seen from Figure 4c,d that the electrical resistance of completely-gelled preserved eggs is small, whereas that of incompletely-gelled preserved eggs is large. When the transient current passes through a completely-gelled preserved egg, the resistance is small. The transient current is not easily blocked and consumed, so it decays slowly.

The resistance of incompletely-gelled preserved eggs is large, which means that the current can easily be blocked and consumed; thus, the current decays fast. The RLC resonance circuit equation obtained using the dielectric method to measure the decay rate of preserved eggs, and the damping oscillation equation for the measurement of the oscillations produced by tapping preserved eggs [12] exhibit the same decay rate. The decay rate for completely-gelled preserved eggs is low, whereas the decay rate for incompletely gel-preserved eggs is high.

### 3.3. Classification of Preserved Egg Gel Based on Impedance

Impedance comprises resistance, which is real, and reactance, which is imaginary. From the Nyquist plot shown in Figure 4d, it can be seen that, for incompletely-gelled eggs, the maximum value of the imaginary reactance in the range 2–300 kHz occurs between approximately 6 and 8 kHz. Therefore, in this study, the impedance, resistance, and reactance at 6 kHz were selected as the characteristic variables for the classification of preserved eggs. A BN classifier was used to classify the quality of the gel in the preserved eggs. The 60 preserved eggs were divided into two types—completely gelled and incompletely gelled—and the gel state of the preserved eggs was classified, as shown in Figure 1. The preserved egg used the impedance, resistance, and reactance as the characteristic classification variables, as given in Table 2 and Table 3. The training results are given in Table 2. The training accuracy obtained using the BN classifier using impedance as the characteristic variable was 95%. The training accuracy obtained using the resistance and reactance as characteristic variables was 96.7%.

Another batch of 116 preserved eggs was used to test the trained BN classification model. The classification accuracy obtained using this batch of eggs was 70.7% for the BN model, which was trained using impedance as the characteristic variable. The classification model trained with resistance and reactance as the characteristic variables achieved a classification accuracy of 81.0%. When the decay rate at 6 kHz was used as the characteristic variable, as given in Table 3, the classification accuracy was 81.9%. Therefore, if the impedance alone is used for classification and prediction, a large number of misjudgments will occur, and some completely-gelled eggs will be classified as incompletely-gelled eggs. The classification results are given in the confusion matrix in Table 4. Overall, the use of the BN classifier with resistance and reactance as the characteristic variables produced the best classification accuracy.

The oscillations of the gelled preserved egg can be felt by the palm using the traditional finger tapping. At the same time, this approach does not work for non-gelled preserved eggs. At an actual factory, workers may tap several thousand preserved eggs per day with their fingers. As the working time increases, the sensitivity of the manual tapping detection will decrease. During the testing process, personal experience and sensations will affect the determination of the gelling and non-gelling of preserved eggs, and there will be differences. The proposed method allows the exclusion of human factors, and a relatively higher detection accuracy compared with the traditional tapping method. The proposed method is different from that of Chen et al. (2011). More specifically, Chen et al. (2011) proposed the use of a plastic stick-tapping method to determine the quality of the gelling in preserved eggs. In their method, the shell was not removed, and the accelerometer was used to measure the oscillations in the eggs [12]. The authors demonstrated that the detection accuracy of gelled and non-gelled preserved eggs could reach 96.7%. The dielectric impedance method employed parallel plate capacitors for the detection. In particular, the parallel plate electrodes touched the equator of the preserved egg. Thus, the signal may be affected by the egg yolk and air factors. It was mainly measured around the equator’s gel condition; moreover, it was difficult to detect the gel condition of the small end of the preserved egg in detail. Therefore, misjudgment occurred, and the detection accuracy was lower than that of Chen et al. [12].

## 4. Conclusions

In this study, preserved eggs were classified as being either completely- or incompletely-gelled based on their real resistance and imaginary reactance, which are the two components of impedance, and using machine learning. This method—based on dielectric impedance measurements—is simple, cheap, and accurate. The measured resistance and reactance within the frequency range of 2–300 kHz were plotted on a Nyquist diagram, and curve fitting was performed in order to obtain the equivalent circuits for completely- and incompletely-gelled preserved eggs. The results of the decay rate obtained by tapping oscillation or using an RLC resonance circuit are similar. The decay rate for completely-gelled eggs was low, whereas the decay rate for incompletely-gelled eggs was high. Using both resistance and reactance as the variables, a BN achieved a classification accuracy of 81.0%, which was greater than that obtained using only impedance used as the variable. The nondestructive dielectric technique used in this research provides an objective and simple way of determining the quality of preserved egg gel, and it proves that dielectric impedance can be used to classify preserved egg gel. In the future, the use of dielectric measurement techniques in automatic systems for the inspection of preserved eggs will be possible.

## Figures and Tables

**Table 1:** Confusion matrix for the preserved egg classification results.

		Predicted State	
	State	Gelled	Non-Gelled
**Actual state**	Gelled	TP	FN
	Non-gelled	FP	TN

**Table 2:** Training results obtained for the BN classifier using the impedance, resistance, and reactance at 6 kHz, and the decay rate at 6 kHz as the characteristic variables.

Variables	Kappa Statistic	Training Accuracy (%)	Precision (%)	Sensitivity (%)
**Impedance (6 kHz)**	0.90	95.0	96.6	93.3
**Resistance (6 kHz), Reactance (6 kHz)**	0.93	96.7	96.7	96.7
**Decay rate (6 kHz)**	0.90	95.0	91.0	100

**Table 3:** Test results for the BN classifier using the impedance, resistance, and reactance at 6 kHz, and the decay rate at 6 kHz obtained using a trained BN classifier.

Variables	Kappa Statistic	Testing Accuracy (%)	Precision (%)	Sensitivity (%)	AUC ROC
**Impedance (6 kHz)**	0.40	70.7	80.0	51.0	0.70
**Resistance (6 kHz), Reactance (6 kHz)**	0.62	81.0	81.1	78.2	0.88
**Decay rate (6 kHz)**	0.64	81.9	75.8	90.9	0.82

**Table 4:** Confusion matrix for the real classification performed after training the BN classifier.

Variables			Predicted State	
			Gelled	Non-Gelled
**Impedance (6 kHz)**	**Actual state**	**Gelled**	28	27
		**Non-gelled**	7	54
**Resistance (6 kHz), Reactance (6 kHz)**		**Gelled**	43	12
		**Non-gelled**	10	51
**Decay rate (6 kHz)**		**Gelled**	50	5
		**Non-gelled**	16	45
